# Prevalence and factors leading to unemployment in MS (multiple sclerosis) patients undergoing immunomodulatory treatment in Poland

**DOI:** 10.1371/journal.pone.0194117

**Published:** 2018-04-10

**Authors:** Dorota Koziarska, Joanna Król, Danuta Nocoń, Przemysław Kubaszewski, Teresa Rzepa, Przemysław Nowacki

**Affiliations:** 1 Department of Neurology, Pomeranian Medical University, Szczecin, Poland; 2 Institute of Psychology, University of Szczecin, Szczecin, Poland; 3 University of Social Sciences and Humanities of Poznań, Poznań, Poland; Charite Universitatsmedizin Berlin, GERMANY

## Abstract

Multiple Sclerosis (MS) is the most common, primary neurogenic cause of disability among young adults. We investigated demographic and clinical factors associated with unemployment on the example of 150 MS patients receiving immunomodulatory treatment in Poland. This study was based on clinical evaluation and collection of self-reported questionnaires, with an attention to self-motivation, severe fatigue and moderate disability. Patients who were unemployed (40% of all patients) had a mean disease duration of almost 5 years. Older (p<0.001), less educated (p = 0.007) and more severely disabled patients (p<0,001) were most likely to be unemployed. Moderate disability (OR = 11.089 95% CI: 4.11–34.201, p<0,001), severe fatigue (OR = 2.625 95% CI: 1.02–6.901, p = 0,046) and lower level of self-motivation (KNS) (OR = 0.947, 95% CI: 0.896–0.006, p = 0.042) were independently associated with unemployment.

## Introduction

### Diagnosis and therapeutic landscape of MS

Revised diagnostic criteria for MS complemented by the results of magnetic resonance imagining (MRI) tests allow for a breakthrough in management of this incurable disease [1]. Clinical diagnosis can be accelerated, and early introduction of immunomodulatory therapy constitutes grounds for prevention of the subsequent consequences of the disease. Continuous treatment of MS has been present and widely available for more than 20 years. Immunomodulatory treatment changes the natural course of the disease and, ideally, stops its progression [2,3,4]. Nowadays, there are a number of therapeutic options available for patients, particularly in the relapsing-remitting course of MS (RR-MS). Selection of the best therapeutic strategy depends on the disease activity. In Poland, therapeutic modalities relying on the use of immunomodulatory injections are widely used and constitute a foundation of treatment.

### Socio-economic impact of MS

According to “Ready to Work” Report of 2011, 80% of patients leave employment within 15 years after the diagnosis [5]. More than 4,600 MS patients from over 100 countries considered the possibility of continuing education and employment as the most important issue for young MS patients [6]. Chronic diseases negatively affect the patients’ productivity. Over the course of the disease, the situation is further aggravated by the changing employment status of the caregivers. Half of the caregivers are forced to modify their professional life in order to provide care to the family members affected by MS [5]. In this respect, MS is one of the most cost-intensive diseases [7,8,9].

### Predictors of employment in MS patients

There are factors known from previous studies that facilitate staying in employment such as: higher educational attainment, low severity of fatigue, lower level of physical disability and cognitive disorders [10,11,12,13]. However, it should be noted that therapeutic strategies that prevent from uncontrolled disease progression should be considered as one more predictor of employment [14]. It was documented in the 11-year-long observation of early beta-interferon treatment—the BENEFIT trial (Betaferon treatment in newly diagnosed MS), that 73% of patients undergoing continuous treatment were in employment. This result accounts for most of the study group, as 81% of the patients were professionally active at the introduction of the treatment [15].

### Role of self-motivation in employment status of MS patients

Only a few studies concern the influence of personality or self-motivation as an independent factor for staying in employment [16,17,18,19]. Therefore, the present study includes the assessment of the hope for success among MS patients–the notion of believing in having a strong will and in one’s ability to find solutions [20,21]. The possibility to assess motivation, including the subjective as well as objective evaluation of factors which facilitate being professionally active, provides a broad view on the issue of employment, from the perspective of both the patient and the therapist.

## Materials and methods

MS patients under care of the regional outpatient Centre for Demyelinating Diseases in Szczecin, Poland. Out of 340 patients from our data base, fulfilling the diagnostic criteria of McDonald [1] and classified according to disease course, 210 responded to our invitation to take part in the study (62%). From among the volunteering patients, 40 were excluded due to depression which was considered to be a known factor limiting professional activity. Due to the profile of injection immunomodulatory drugs, the assessment of potential depression is an integral part of medical supervision. There was no depression score used, diagnosis was based on a psychiatric consultation and/or indications for antidepressant treatment during the last 6 months. Depression was an exclusion criterion adopted for the purpose of analysing the other, less obvious factors affecting termination of employment. In the case of 20 patients the investigated questionnaires assessing level of fatigue and/or subjective perceived cognitive deficits and/or level of self-motivation were incompletely filled. Therefore, we excluded them for further analysis. The total number of 150 clinically stable patients over 18 years of age took part in the study conducted between September 2015 and January 2016. Most patients in the study group (86%) fulfilled the diagnostic criteria for relapsing-remitting MS (RR-MS) n = 130. Patients with progressive MS were in minority: secondary progressive (SP-MS; n = 15; 10%), primary progressive (PP-MS n = 5; 4%).

To assess the clinical condition and self-motivation of the patients as factors which influence professional activity, we divided the study group into professionally active (employed) and inactive (unemployed) cohorts. The patients provided information regarding their employment status by selecting one of the following options: employed either full-time or part-time, or not employed. An account has also been taken of whether a patient is a labourer or a white-collar worker, including being professionally active while still at school or university. In the unemployed group, the relationships between employment status and disability due to the disease (disability pension, rehabilitation benefit), age (retirement) or personal circumstances (being a dependent family member without disability) were analysed.

All the participants completed self-reported questionnaires. S1 Appendix

We collected structured demographic interviews and data on the current status of treatment with disease-modifying therapy (DMT). Neurological disability was rated by a single neurologist (DK) with the use of the Expanded Disability Status Scale (EDSS). Patients with a result of up to 3.0 are fully ambulatory, with only a mild disability. Above the score of 3.0, disability can be termed moderate and it influences daily activities. [22]. Fatigue was assessed by the Fatigue Severity Scale (FSS). The cut-off score over 4.0 indicates severe fatigue [23,24]. Subjective intellectual problems were identified with the Perceived Deficits Questionnaire 5-item version (PDQ5) which analyses the influence of cognitive problems in daily living activities. Higher scores indicate greater cognitive problems. The minimum is 0 and the maximum is 20. There is no cut-off score known [23]. The analysis of self-motivation was done by the Polish adaptation of KNS (Hope for Success Questionnaire). The result is a sum of points reflecting the level of hope for success. However, apart from providing a valid indicator of the level of hope, i.e. the general result, the questionnaire allows for analysis of two components of hope: solution-finding skills and strong will [21].

All subjects gave their informed consent for inclusion before they participated in the study. The study was conducted in accordance with the Declaration of Helsinki, and the protocol was approved by the Ethics Committee of the Pomeranian Medical University in Szczecin (KB-0012/137/15). The follow-up analysis is planned as a multi-centre study.

### Statistical analysis

The statistical analysis was performed using R (The R Project for Statistical Computing)- version 3.2.3. In descriptive statistics the following tests were used: Fisher’s exact test to establish differences in proportions, T-test to establish differences in quantitative data (for normally distributed data), Wilcoxon test to establish differences in quantitative data (for non-normally distributed data), Anderson-Darling test to establish the normality of given quantitative data. In order to determine the influence of main neurological rates (EDSS, FSS, PQD5 and KNS) on unemployment status, the univariate and multivariate logistic regressions were performed.

## Results and discussion

### Employment status

The study group comprised patients aged from 18 to 72 (mean age 40.6 years), with a mean EDSS score of 2.37. 60% (n = 90) of the participants were professionally active. Within this group, 51% (n = 46) performed physical work and 49% (n = 44) were white collar workers, including full-time students and pupils (n = 6). Among professionally active patients, 14 employees were entitled to a retirement or disability pension. The group of professionally inactive patients (n = 60) comprised patients who reached the retirement age (n = 5), were entitled to a disability pension (n = 43), a rehabilitation benefit (n = 3), as well as family dependents (n = 9). Disability pensions or rehabilitation benefits for all the patients were awarded due to MS.

### Demographic variables

Patients professionally inactive were older (p<0.001), with the mean difference from the employed group of 8.19 years. The majority of the participants were female, yet no difference was observed as to remaining in employment. Unemployed patients were less educated (p = 0.007). The number of children was higher in the unemployed group (p<0.01). The employment status was not determined by the place of residence.

### Disease duration and treatment delay

Progressive forms of MS were present in 28% of the unemployed group and led more frequently to unemployment (p<0,01). The patients who left employment had been showing symptoms for more years (p = 0.042) and the treatment had been initiated later (p = 0.054). A mean disease duration in the unemployed patients (40% of all patients) was almost 5 years. Although the disease duration was similar in both groups, the unemployed patients were currently less frequently undergoing immunomodulatory treatment (p<0,01). We didn’t find any difference regarding the age at diagnosis (p = 0.08), delay in treatment initiation (p = 0.748) or duration of treatment (p = 0.879) between both analysed groups—Table 1.

**Table 1:** Socio-demographic and clinical characteristic of MS patients.

Variable	Employed (n = 90)	Unemployed (n = 60)	p value
Age (Mean(SD))	36.51(9.49)	44.7(12.62)	<0,001
Gender (female/male))	58/32	40/20	0.862
Total years of education (Mean(SD))	14.77(3.1)	13.41(2.57)	0.007
Number of children (0)%	37/41.1	11/18.33	-*
Number of children (1)%	32/35.56	17/28.33	0.203
Number of children (2)%	15/16.67	20/33.33	<0.01
Number of children (3 and more)%	6/6.67	12/20.01	<0.01
Country/City n	16/74	10/50	0.86
MS Course RR/SP/PP [n]	87/3/0	43/12/5	<0.01
First symptoms to now in years (Mean(SD))	7.78(7.16)	12.03(10.86)	0.042
Age at disease onset in years (Mean(SD))	28.8(8.66)	32.78(13)	0.128
Age at diagnosis in years (Mean(SD))	31.76(9.16)	37.54(10.81)	0.08
Age at initiation of treatment in years (Mean(SD))	33.93(9.16)	37.54(10.81)	0.054
Disease duration (Diagnosis to now in years) (Mean(SD))	4.17(3.77)	4.93(4.52)	0.46
DMT treatment yes/no [n]	83/7	41/19	<0.01
DMT treatment in years (Mean(SD))	2(1.75)	2.1(2.01)	0.879
Delay in treatment in years (Mean(SD))	2.12(3.18)	2.88(4.32)	0.748

SD: standard deviation, DMT: disease modifying therapy, RR: relapsing–remitting SP: secondary progressive, PP: primary progressive, p value represents the statistical difference between the unemployed and employed MS patients, p-value * number of children 0 as a reference point

Taking into account young population with major clinical presentation of RRMS and rather short disease duration, we further analysed the clinical differences between both groups.

### Disease severity and self-motivation

The level of disability, considered to be indicative of disease advancement, was significantly higher in patients with progressive types of MS with mean EDSS score 5.425. Whereas patients with RR-MS were characterised by lower level of disability with mean EDSS score 1.719. The average EDSS score for the unemployed MS patients was twice as high as that of the employed (p<0.001). The analysis of FSS showed a higher level of fatigue in the unemployed group (p<0.001). More severe subjective cognitive problems in daily living (PDQ5) were found in the unemployed MS patients (p = 0.004). Moreover, motivation measured using the KNS scale was less prominent in this group (p = 0.003)—Table 2.

**Table 2:** Main predictors of unemployment.

Variable	Employed (n = 90)	Unemployed (n = 60)	p-value
EDSS (Mean(SD))	1.57(0.98)	3.18(2.03)	<0,001
FSS (Mean(SD))	3.46(1.42)	4.74(1.64)	<0,001
PQD5 (Mean(SD))	5.19(4.31)	7.48(4.82)	0.004
KNS(Mean(SD))	49.34(7.38)	44.63(10.47)	0.003

EDSSS: Expanded Disability Status Scale, FSS: Fatigue Severity Scale, PQD5: Perceived Deficits Questionnaire 5-items version, KNS: Hope for Success Questionnaire

### Factors influencing employment

Patients who withdrew from employment had higher EDSS scores. The cut-off EDSS > 3 was established with OR 13.227, (p<0.01). Also the progressive type of disease was a strong predictor of unemployment OR 11.465, (p<0.01). Similarly, the patients suffered from more severe fatigue, the cut-off FSS>4 was connected with OR 4, (p<0.01), they showed lower self-motivation (KNS) (OR = 0.941, p<0.01). Intellectual deficits experienced by these patients interfered to a greater extent with daily life activities (PDQ5) (OR = 1.115, p<0.01) Table 3.

**Table 3:** Univariate logistic regression analysis of factors influencing employment.

	OR	95% CI	p -value
Row EDSS	1.965	1.537–2.615	<0.01
EDSS 2–3	0.512	0.139–1.518	0.262
EDSS>3	13.227	5.221–38.741	<0.01
Disease type SP/PP	11.465	3.613–51. 034	<0.01
PQD5	1.115	1.038–1.203	<0.01
KNS	0.941	0.903–0.977	<0.01
FSS>4	4	2.024–8.127	<0.01

EDSSS: Expanded Disability Status Scale, FSS: Fatigue Severity Scale, PQD5: Perceived Deficits Questionnaire 5-items version, KNS: Hope for Success Questionnaire.

Multivariate analysis revealed that moderate level of neurological disability (EDSS>3) (OR = 11.089, p< 0.01), more severe fatigue (FSS>4) (OR = 2.65, p = 0.048) and less present self-motivation (KNS) (OR = 0.947, p = 0.042) were independently associated with unemployment Table 4.

**Table 4:** Multivariate logistic regression analysis of factors influencing employment.

	OR	95% CI	p-value
FSS >4	2.625	1.02–6.901	0.046
EDSS >3	11.089	4.116–34.201	<0.01
PQD5	1.014	0.9–1.138	0.821
KNS	0.947	0.896–0.006	0.042

EDSSS: Expanded Disability Status Scale, PQD5: Perceived Deficits Questionnaire 5-items version, KNS: Hope for Success Questionnaire

The employment situation of a young adult diagnosed with an incurable disease has been widely discussed in the literature on the subject in many countries [25,26,27,28] Therefore, this paper provides data on factors determining unemployment among MS patients in Poland. To our knowledge, this is the first report about demographic and clinical factors leading to unemployment in Polish MS patients who undergo immunomodulatory therapy. The report indicates that demographic factors such as higher age at the moment of participation in the research study and on the onset of first symptoms have a negative influence on the employment status. We found the number of children to be significantly higher among unemployed women. It may potentially indicate a decision to withdraw from professional activity due to having many children, not because of severity of symptoms or disease advancement only.

Despite higher educational attainment, only some of the participants were in employment. Less educated MS patients are more likely to withdraw from employment. These findings are consistent with the results obtained in other countries [29,30,31]. It is often the case that patients suffering from MS take less well-paid jobs so as to stay in employment [8,32], while their healthy counterparts enjoy their career opportunities with the perspective of an income increase [9].

In terms of the disease duration, 40% of the patients from the cohort were unemployed approximately after 5 years from the diagnosis despite the fact that most of them were in treatment. Flachenecker et al.[26] reported the same proportion of unemployment even after 3 years of disease duration.

The most significant determinant of unemployment in our study was EDSS >3 which increased the possibility of unemployment by 13 times even though the patients were not severely disabled, having a mean EDSS score of 2.37 From the cut of EDSS 3.0, day-to-day functioning is gradually limited and the conversion to progressive disability became more probable. Therefore the higher disability levels as well as being diagnosed with progressive types of MS, increase the risk of unemployment by several times. This correlation is a natural result of limited abilities of the patients both factors are strongly interlinked.

MS, as a chronic central nervous system (CNS) disease, affects not only motor functions, which is well represented by EDSS scores, but also leads to impaired cognition and general fatigue [24,33, 34,35,36].

The studies by O’Connor et al. [37] and Simmons et al. [8] indicate that chronic fatigue, impaired memory and concentration are important factors limiting employment. In our study, more than 66% of the unemployed patients experienced fatigue to the point of limiting activities of daily living (FSS score >4.0). This factor increases the risk of unemployment by 4 times. Additionally, self-reported problems with concentration, assessed with the PDQ5 scale, were more prominent in the unemployed group.

The analysis of independent factors leading to unemployment identified the following factors: moderate disability, severe fatigue and lower level of self-motivation.

The newly analysed factor of self-motivation seems to have been neglected in terms of its relation to long term influence on employment. In our cohort there were 14 still professionally active patients who were entitled to a retirement or disability pension. The fact that these patients continued to be employed while the majority withdrew from employment is worth studying.

Having low hopes for success is associated with an attitude of avoidance, i.e. passive attitude [38,39]. Motivation to achieve success, analysed in this paper according to Snyder, constitutes a learned thinking pattern which serves as an adaptive mechanism to change the life situation, allowing the person to be more flexible and persistent [40]. Moreover, it is linked with high self-esteem and a higher life satisfaction level, which provides more flexible approach to difficult life events and facilitates finding alternative solutions.

Assessment of self-motivation may allow identification of the patients who engage in preventive actions and show a greater perseverance in terms of treatment adherence. Higher level of hope for success corresponds with better adaptation strategies, higher level of general self-esteem and the ability to endure physical pain [41,42].

Both physical disability as well as non-motor symptoms of MS greatly affect motivation and work ability of patients. The focus of interdisciplinary preventive therapeutic teams should be on establishing long-term motivation and non-motor MS symptoms.

## Conclusions

The result of our study indicates high frequency of unemployment among MS patients in Poland, despite the immunomodulatory treatment. Ability to work was mainly influenced by increasing disability over the course of the disease. Not only motor symptoms, but also fatigue and cognitive deficits contribute to early unemployment. The newly evaluated factor of self-motivation complements the previously known factors. The relatively small sample size could be a limiting factor of the present study. Prospective evaluations are planned.

## Supporting information

S1 Appendix Employment status questioner. (PDF)

S1 Table Database. (XLSX)
