# Supplementary material for: Prevalence and factors leading to unemployment in MS (multiple sclerosis) patients undergoing immunomodulatory treatment in Poland
Source: PLoS One. 2018 Apr 10;13(4):e0194117. doi: 10.1371/journal.pone.0194117 (PMC5892902; doi:10.1371/journal.pone.0194117)
Supplement: S1 Appendix — (PDF) [file pone.0194117.s001.pdf]

Employment Status

GENDER: **MAN/FEMALE**

Age (date of birth):

EDUCATION:

□ elementary □ vocational □ secondary □ higher

TOTAL NUMBER OF YEARS IN EDUCATION: ....

MARITAL STATUS:

□ married □ widowed □ divorced □ single

NUMBER OF CHILDREN..........................

PLACE OF RESIDENCE (number of citizens)

□ country □ town up to 25 000 □ town 25 000 – 100 000 □ town more than 100 000

CURRENT EMPLOYMENT STATUS:

□ unemployed

□ employed

WORKING TIME:

□ employed as physical workers

□ full time □ part time □ overtime

□ employed as a white collar or student or pupil

□ full time □ part time □ overtime

WORKING TIME IS MOSTLY DETERMINED BY YOUR:

□ health status □ local labour market □ other

Reason of being unemployed:

□ retirement **Year of qualifying**

□ disability pension **Year of qualifying**

Due to MS **yes/no**

□ rehabilitation benefit **Year of qualifying for the benefit**

□ family dependent **Since**
